# Spatial metabolomics identifies localized chemical changes in heart tissue during chronic cardiac Chagas Disease

**DOI:** 10.1371/journal.pntd.0009819

**Published:** 2021-10-04

**Authors:** Danya A. Dean, Gautham Gautham, Jair L. Siqueira-Neto, James H. McKerrow, Pieter C. Dorrestein, Laura-Isobel McCall

**Affiliations:** 1 Department of Chemistry and Biochemistry, University of Oklahoma, Norman, Oklahoma, United States of America; 2 Laboratories of Molecular Anthropology and Microbiome Research, University of Oklahoma, Norman, Oklahoma, United States of America; 3 Department of Biology, University of Oklahoma, Norman, Oklahoma, United States of America; 4 Skaggs School of Pharmacy and Pharmaceutical Sciences, University of California San Diego, La Jolla, California, United States of America; 5 Center for Microbiome Innovation, University of California San Diego, La Jolla, California, United States of America; 6 Collaborative Mass Spectrometry Innovation Center, University of California San Diego, La Jolla, California, United States of America; 7 Department of Microbiology and Plant Biology, University of Oklahoma, Norman, Oklahoma, United States of America; University of Texas at El Paso, UNITED STATES

## Abstract

Chagas disease (CD), caused by the parasite *Trypanosoma cruzi*, is one of nineteen neglected tropical diseases. CD is a vector-borne disease transmitted by triatomines, but CD can also be transmitted through blood transfusions, organ transplants, *T*. *cruzi-*contaminated food and drinks, and congenital transmission. While endemic to the Americas, *T*. *cruzi* infects 7–8 million people worldwide and can induce severe cardiac symptoms including apical aneurysms, thromboembolisms and arrhythmias during the chronic stage of CD. However, these cardiac clinical manifestations and CD pathogenesis are not fully understood. Using spatial metabolomics (chemical cartography), we sought to understand the localized impact of chronic CD on the cardiac metabolome of mice infected with two divergent *T*. *cruzi* strains. Our data showed chemical differences in localized cardiac regions upon chronic *T*. *cruzi* infection, indicating that parasite infection changes the host metabolome at specific sites in chronic CD. These sites were distinct from the sites of highest parasite burden. In addition, we identified acylcarnitines and glycerophosphocholines as discriminatory chemical families within each heart region, comparing infected and uninfected samples. Overall, our study indicated global and positional metabolic differences common to infection with different *T*. *cruzi* strains and identified select infection-modulated pathways. These results provide further insight into CD pathogenesis and demonstrate the advantage of a systematic spatial perspective to understand infectious disease tropism.

## Author summary

Chagas disease (CD) is a tropical disease caused by the parasite *Trypanosoma cruzi*. CD originated in the Americas but is now found globally due to population movements. CD is transmitted through a triatomine vector, organ transplants, blood transfusions, *T*. *cruzi-*contaminated food and drinks, and congenitally. It occurs in two stages, an acute stage (usually asymptomatic) and a chronic stage. Twenty to thirty percent of chronic stage cases present severe cardiac symptoms such as heart failure, localized aneurysms and cardiomyopathy. Unfortunately, what causes severe cardiac symptoms in some individuals in chronic CD is not fully understood. Therefore, we used liquid chromatography-tandem mass spectrometry to analyze the heart tissue of chronically *T*. *cruzi-*infected and uninfected mice, to understand the impact of infection on the tissue metabolome. We identified discriminatory small molecules related to *T*. *cruzi* infection and determined that regions with the highest parasite burden are distinct from the regions with the largest changes in overall metabolite profile. These locations of high metabolic perturbation provide a molecular mechanism to explain why localized cardiac symptoms occur in CD, particularly at the heart apex. Overall, our work gives insight into chronic cardiac CD symptom development and shapes a framework for novel CD treatment.

## Introduction

Chagas disease (CD) is a parasitic disease caused by the protozoan *Trypanosoma cruzi* and is one of the designated neglected tropical diseases [1]. *T*. *cruzi* is endemic to the Americas and infects 7–8 million people worldwide [1]. An estimated 300,000 infections have been recorded in the United States due to a large Latin American immigrant population and endemic transmission [2–4]. CD is primarily transmitted through triatomine insects of the *Triatoma* and *Rhodnius* genera [2,5]. Non-vectorial modes of transmission involve blood transfusion, transplacental transmission, and food and drink contaminated with *T*. *cruzi* [1]. The *T*. *cruzi* life cycle includes three main stages: epimastigotes, trypomastigotes and amastigotes. *T*. *cruzi* in the insect vector undergoes transformation from trypomastigotes to epimastigotes in the midgut, and then migrates to the hindgut and differentiates into infective trypomastigotes [1]. Upon triatomine defecation on the human host, the infective trypomastigotes enter the host through scratching or rubbing of the bite wound, or through eyes and mucosal surfaces [1]. Following mammalian host cell infection, trypomastigotes differentiate into amastigotes, which proliferate and subsequently transform into trypomastigotes [1].

CD has two disease stages: acute and chronic [1,2,5]. The acute stage, with high parasite load, is usually asymptomatic, or presents with non-specific symptoms (fever, malaise) [1,2,5]. A minority of infected individuals (20–30%) will then progressively develop clinical manifestations of chronic CD, including cardiomegaly, cardiac arrhythmias, apical aneurysms, megacolon, and megaesophagus [2]. In contrast to acute stage CD, chronic CD presents with low to no parasitemia [5]. *T*. *cruzi* infections are treated with either benznidazole or nifurtimox; however, these treatments cause significant adverse effects, to the point that up to 30% of treated individuals fail to complete the full treatment course [6,7].

CD was previously considered to have an autoimmune etiology, but parasite persistence has now conclusively been demonstrated to be required for disease pathogenesis [8]. Along with parasite persistence, chronic pro-inflammatory responses, including cytokine release and CD8+ T cell- mediated cytotoxicity, contribute to tissue damage [9]. A heterogeneity of interacting parasite-host factors, including *T*. *cruzi* strain, load and tissue tropism, host genetic background, and mode of infection, influence the clinical outcomes of the disease [10,11]. However, CD pathogenesis is not yet completely understood [2]. A holistic understanding of the molecular pathways involved in disease progression could help identify new drug development avenues.

Metabolites are the final products of mRNA and protein expression and of protein activity, thus providing information closely linked to phenotype [12]. Metabolic pathways are druggable. They also change dynamically in response to disease [13,14]. As such, an improved understanding of metabolism in CD may lead to new avenues for drug development and CD patient monitoring. Acute *T*. *cruzi-*infection affects *in vitro* and *in vivo* host metabolic pathways, including decreasing mitochondrial oxidative phosphorylation-mediated ATP production [9,15–17]. In addition, acutely *T*. *cruzi*-infected mice heart tissue and plasma showed significant changes in certain metabolic pathways, such as glucose metabolism (glucose levels elevated in heart tissue and lowered in plasma over time), tricarboxylic acid cycle (TCA) (decrease in select TCA metabolites in the heart tissue and a decrease in all detected TCA metabolites in plasma), lipid metabolism (increased long-chain fatty acids in the heart tissue and decreased long-chain fatty acids in plasma), and phospholipid metabolism (high accumulation of phosphocholine precursor metabolites in the heart) [15]. Prior analysis of hearts from acutely infected mice also showed that cardiac metabolite profiles reflected disease severity, with changes in cardiac acylcarnitines and glycerophosphocholines predictive of acute infection outcome [9]. Metabolomic analysis of chronic CD has been limited to serum and gastrointestinal tract samples [18,19]. Serum analysis demonstrated significant changes in amino acid and lipid metabolism, particularly acylcarnitines, sphingolipids, and glycerophospholipids [19]. Analysis of GI tract samples observed persistent metabolic perturbations in the oesophagus and large intestine in chronic CD, including infection-induced elevation of acylcarnitines, phosphatidylcholines and amino acid derivatives [18]. However, metabolic changes in the heart may differ from those in the circulation or in the GI tract [15]. It is therefore essential to perform metabolomic analysis of tissues collected from the heart in chronic CD.

Many sudden fatalities due to chronic cardiac CD are often attributed to apical aneurysms which occur at the bottom of the heart [20,21]. Importantly, parasite load is low, spatially heterogeneous and poorly correlated to the magnitude of tissue damage in chronic CD, including in clinical samples [22,23], indicating possible spatial disconnect between CD-induced metabolic alterations and tissue parasite load. We therefore focused on liquid chromatography-tandem mass spectrometry-based metabolomic analysis of transversely sectioned hearts from mice chronically infected with *T*. *cruzi* strains CL and Sylvio X10/4. These samples were previously collected but only previously analyzed with regards to parasite load and differences in overall metabolome between heart apex and heart base (infected and uninfected samples combined), without characterizing the impact of chronic infection on the heart metabolome [9]. In contrast, this study focuses on metabolic changes associated with chronic (90 and 147 days) *T*. *cruzi* strains CL and Sylvio X10/4 infection, compared to matched uninfected controls. Overall, we observed significant localized chemical differences associated with infection, with a disconnect between parasite localization and overall positional metabolic perturbations. Our data also showed infection-induced variations in acylcarnitine and glycerophosphocholine chemical families.

## Methods

### Ethics statement

All vertebrate animal studies were performed in accordance with the USDA Animal Welfare Act and the Guide for the Care and Use of Laboratory Animals of the National Institutes of Health. The protocol was approved by the University of California San Diego Institutional Animal Care and Use Committee (protocol S14187).

### *In vivo* experimentation

All *in vivo* experimentation and sample collection were conducted and previously reported in [9]. Briefly, n = 5 five-week-old male C3H/HeJ mice (Jackson Laboratories) were infected with 1,000 *T*. *cruzi* strain CL trypomastigotes, with n = 7 matched uninfected controls; both groups were euthanized 90 days post-infection. N = 5 five-week-old male C3H/HeJ mice (Jackson Laboratories) were infected with 1 million *T*. *cruzi* strain Sylvio X10/4 trypomastigotes, with n = 5 matched uninfected control mice. N = 4 of these *T*. *cruzi* Sylvio X10/4-infected animals survived to the endpoint, at 147 days post-infection, when they were euthanized along with matched controls. Euthanized mice were perfused with phosphate-buffered saline to clear any circulating trypomastigotes. The hearts were removed, transversely sectioned into four sections from the base to the apex of the heart (sections A to D in text below) and snap-frozen using liquid nitrogen, for a total of n = 4 sections per animal. Each section was then used in its entirety for metabolite extraction and LC-MS analysis, so that we have complete and consistent spatial coverage of the heart tissue.

Although sample collection was reported in McCall *et al [9]*, the authors only studied the acute impact (12 days post-infection) of Chagas disease on mouse hearts. This study, in contrast, examines the impact of infection on the heart samples extracted on days 90 and 147 post-infection (chronic stage).

qPCR was performed in McCall *et al* [9]. Briefly, a Quick-DNA universal kit from Zymo Research was used to extract DNA from homogenized heart tissue sections, and a nanodrop was used to quantify DNA. 180 ng was used for qPCR analysis. ASTCGGCTGATCGTTTTCGA and AATTCCTCCAAGCAGCGGATA primers were used to amplify parasite satellite DNA region [24] and TCCCTCTCATCAGTTCTATGGCCCA and CAGCAAGCATCTATGCACTTAGACCCC to amplify host TNFα [25]. The following parameters were used for amplification: initial denaturation for 10 min at 95°C, then 40 cycles of denaturation (95°C for 30s), annealing (58°C for 60s), and extension (72°C for 60s). PCR product formation was confirmed through melting curve analysis. Parasite burden in each heart section was determined using a standard curve developed from samples extracted from mouse heart tissue spiked with 2 x 10^7^ *T*. *cruzi* epimastigotes [9].

### Metabolite extraction and UHPLC-MS/MS

The two-step procedure for metabolite extraction was conducted as described in McCall *et al* [9], with “aqueous” and “organic” extracts referring to the metabolites recovered from the first 50% methanol extraction and the second 3:1 dichloromethane-methanol extraction, respectively. Dried samples were obtained and resuspended in 50% methanol spiked with sulfadimethoxine as internal standard. A checkmix solution with 6 known molecules was also run at the beginning and end of LC-MS analysis to monitor instrumental drift and showed only minor retention time shifts (S1 Fig). Both extracts were analyzed separately and were randomized according to infection type as well as position to prevent sample bias. Thermo Scientific UltiMate 3000 Ultra High Performance Liquid Chromatography was used to analyze samples using a 1.7 μm C8 (50 x 2.1 mm) UHPLC column (Phenomenex) equipped with a C8 guard cartridge (Phenomenex). Chromatography was done with water + 0.1% formic acid (mobile phase A) and acetonitrile + 0.1% formic acid (mobile phase B), at a 0.5 mL/min flow rate (7.00 min aqueous extract and 10.5 min organic extract) with a 40°C column temperature. LC gradient for aqueous extract is as follows: 0–1 min, hold at 2% B; 1–1.5 min, increase to 40% B; 1.5–4 min, increase to 98% B; 4–5 min, hold at 98% B; 5–6 min, decrease to 2% B; 6–7 min, hold at 2% B. LC gradient for organic extract is as follows: 0–1 min, hold at 2% B; 1–1.5 min, increase to 60% B; 1.5–5.5 min, increase to 98% B; 5.5–7.5 min, hold at 98% B; 7.5–8.5 min, decrease to 2% B; 8.5–10.5 min, hold at 2% B.

MS/MS detection was conducted on a Maxis Impact HD QTOF mass spectrometer (Bruker Daltonics) [9]. Electrospray ionization was used to generate ions and MS spectra obtained in positive mode only. ESI-L Low concentration Tuning Mix (Agilent Technologies) was used for daily instrument calibration and Hexakis(1H,1H,3H-tetrafluoropropoxy)phophazene (Synquest Laboratories), *m/z* 922.009798, was used throughout the analysis as internal calibrant (lock mass).The following instrumental parameters were used for UHPLC-MS/MS: runtime: 0 to 7 min (aqueous extract), 0 to 10.5 min (organic extract); polarity: positive; exclusion: on; nebulizer gas pressure: 2 bar; capillary voltage: 4,500 Volts; ion source temperature: 200°C; dry gas flow: 9.0 L/min, spectra rate acquisition: 3/sec; TopN: 7 (aqueous extract), 10 (organic extract); mass range: 80–2,000 *m/z*; active exclusion: after 4 spectra and release after 30s; ramped collision-induced dissociation energy: 10–50 eV. Mass ranges with common contaminants and lock masses were excluded (*m/z* 123.59–124.59, 143.50–144.50, 159.47–160.47, 182.49–183.49, 216.61–217.61, 309.83–310.83, 337.50–338.50, 359.50–360.50, 622.00–622.05, 921.50–925.50).

### LC-MS/MS data analysis

Data processing was performed as previously reported using Optimus, July 21, 2016 version [9,26,27]. Optimus data processing parameters are as follows: (i) LC-MS feature detection: *m/z* tolerance: 20.0 ppm; noise threshold: 1000; half of MS/MS isolation window: 2.0 Da. (ii) Advanced FD settings: commom_chrom_fwhm: 20; common_chrom_peak_snr: 2.0; mtd_reestimate_mtd_std: enabled; epd_width_filtering: off; epd_min_fwhm: 1.5; edp_max_fwhm: 25.0; ffm_report_summed_ints: Enabled. (iii) Filter features: minimum occurrence rate: 0.01. (iv) Missing feature intensities: Enable imputation of missing features: Enabled. (v) Normalize features: Enable feature normalization using internal standards (sulfadimethoxine injection control): enabled.

Data was normalized to sulfadimethoxine internal standard peak, followed by total ion current (TIC) normalization in R. Random forest analyses were performed separately on the aqueous extract feature table and the organic extract feature table (both total ion current (TIC)-normalized), with both results jointly summarized in figures and tables. Principal coordinate analysis (PCoA) was performed on total ion current (TIC) normalized MS1 feature data table using the Bray-Curtis-Faith dissimilarity metric using QIIME1 [28], for both organic and aqueous extractions combined. The three-dimensional PCoA plots were visualized in EMPeror [29]. Three-dimensional data visualization was performed using ‘ili’ (http://ili.embl.de/) [27] using a three dimensional heart model from 3DCADBrowser.com (http://www.3dcadbrowser.com/).

Global Natural Products Social Molecular Networking (GNPS) was used to perform molecular networking according to the following parameters: precursor mass tolerance: 0.02 Da; fragment ion mass tolerance: 0.02 Da; cosine score: 0.7; minimum matched fragment ions: 4; search analogs: do search; network TopK: 10; maximum connected component size: 100; minimum cluster size: 2; score threshold: 0.7; library search min. matched peaks: 4; max. analog search mass: 100; filter precursor window: filter; filter peaks in 50 Da window: filter; filter below Std. Dev: 0.0; min. peak intensity: 0.0; filter library: filter; filter spectra from G6 as blanks before networking: don’t filter [30].

Metabolite annotation was based on selected libraries in the GNPS infrastructure: GNPS-COLLECTIONS-MISC, GNPS- EMBL-MCF, GNPS-FAULKNERLEGACY, GNPS-LIBRARY, GNPS-NIH-CLINICALCOLLECTION1, GNPS-NIH-CLINICALCOLLECTION2, GNPS-NIST14-MATCHES, HMDB, MASSBANK, MASSBANKEU, PNNL-LIPIDS, MONA. Direct MS2 spectral matches to these libraries enable metabolomics standard initiative level 2 annotation confidence [31]. All spectral matches were visually inspected and MS2 fragment annotation was performed using CFM-ID [32,33], HMDB [34,35] and LipidMaps [36]. Cytoscape 3.7.0. was used to visualize the molecular networks [37]. Molecular networks were used to annotate metabolites without a direct match to GNPS libraries, using sub molecular network chemical families and annotation propagation [30], enabling metabolomics standard initiative level 3 annotation confidence [31]. Given the fragmentation pattern for glycerophosphocholines (PCs) under our instrumental conditions, we cannot distinguish based on MS2 pattern or *m/z* between PC isomers, such PC O-16:0/18:1 and PC O-12:0/22:1. Therefore we reported both library spectral matches and all possible other possible annotations based on LipidMaps nomenclature [36]. Lastly, annotations were filtered based on plausibility vis-à-vis the observed retention time.

Random forest analysis was performed in Jupyter Notebook using R with the number of trees set to 500. Random forest classifier cutoff was based on ranked variable importance score of differential metabolites >1.3. FDR-corrected Mann-Whitney p<0.05 for all positions was used as an alternate method to determine significant metabolite differences. Venn diagrams were used to visualize the unique and common metabolites differential between CL and Sylvio X10/4 infection, compared to uninfected samples, based on heart segment positions, random forest classifier for all positions, or FDR-corrected Mann-Whitney p<0.05 for all positions, using http://bioinformatics.psb.ugent.be/webtools/Venn/. Effect size calculations were performed on total acylcarnitine and glycerophosphocholine levels in heart section A using Hedges’ g, via https://www.socscistatistics.com/effectsize/default3.aspx.

PCs were selected through the following steps: 1) Collect all metabolite features annotated as PCs through matching to GNPS reference libraries, for each extract (including matches with identical *m/z* to reference libraries and analog matches, with mass differences representing for example PCs with different chain lengths or degree of saturation compared to reference libraries). 2) Extend these annotations using molecular networking, collecting all metabolites with shared fragmentation patterns to these library matches. This ensures that all candidate PCs with quality MS2 fragmentation patterns are selected. 3) Verify that the MS2 spectra contain the PC diagnostic peaks of *m/z* 86.10 (N,N,N-Trimethylethenaminium), *m/z* 125.00 (2,2-Dihydroxy-1,3,2-dioxaphospholan-2-ium) and *m/z* 184.08 (phosphocholine). 4) Verify *m/z* presence in LipidMaps [36]. 5) Subset feature abundance table to these *m/z* and retention times, to obtain a new feature table with the peak area of PCs in our samples. Specifically, there were a total of 396 PCs where 81 were short (400–599.99 *m/z*), 207 were mid-length (600–799.99 *m/z*), and 108 were long (>800 *m/z*). A similar process was used to sub-select acylcarnitines, monitoring presence of the 3-carboxyallylium +CH_2_-CH = CH-COOH diagnostic peak at *m/z* 85.03). There were a total of 84 acylcarnitines, which were assigned to short- (C2-C4), mid- (C5-C11) and long-chain (C12 and longer) categories using LipidMaps [36].

## Results

The purpose of this study was to compare the metabolic impact of chronic CD in the mouse heart between divergent *T*. *cruzi* strains and between cardiac regions. To do so, we analyzed previously-collected positive mode LC-MS/MS data [9]. While this prior study focused on the impact of acute infection (12 days post-infection in mice) on the cardiac metabolite profile, here we specifically focused on the impact of 90 and 147day infection (chronic stage CD in mice) on the cardiac metabolite profile, which had not been studied before.

**Fig 1:**
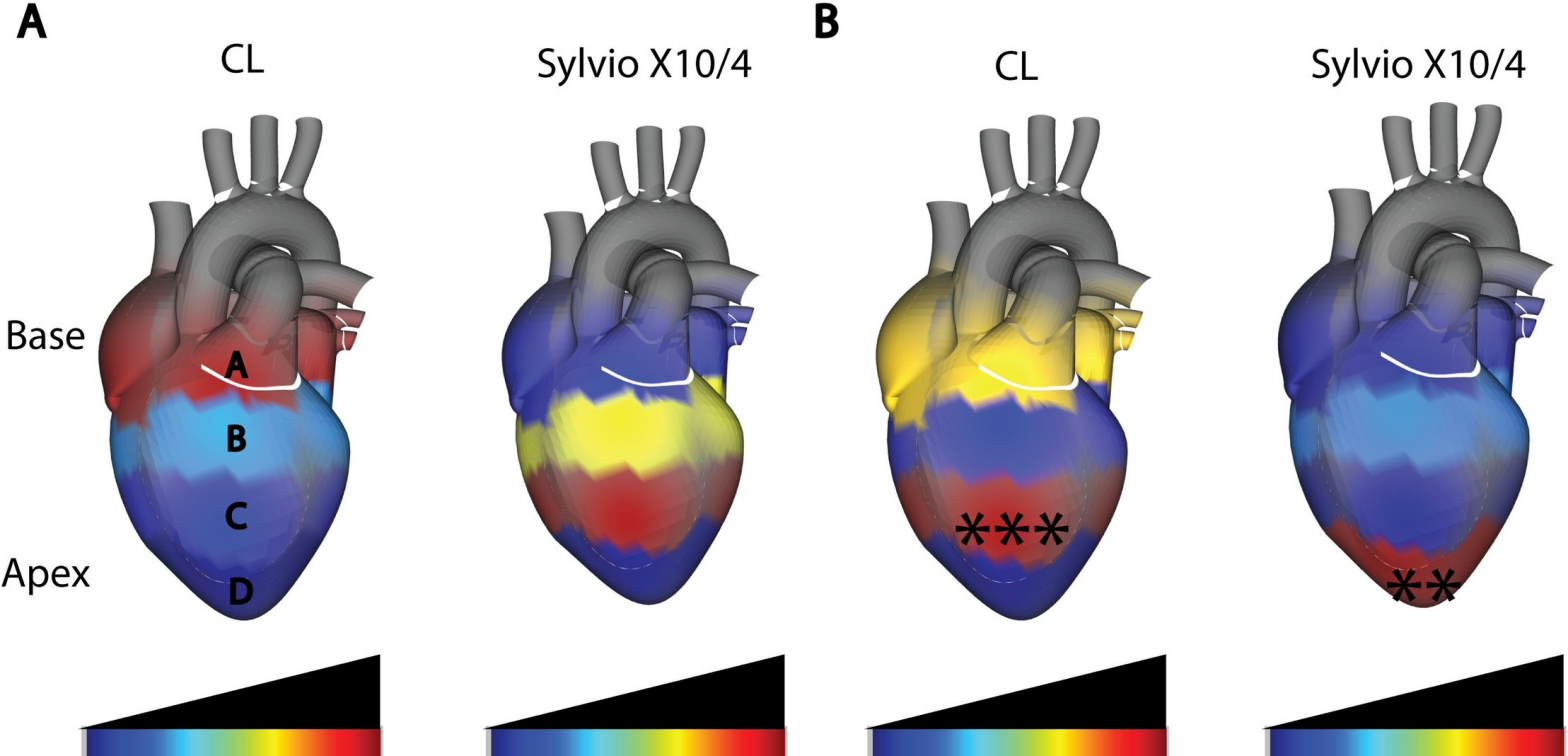
Disconnect between sites of parasite persistence and metabolic alterations in chronic cardiac CD. (A) Median cardiac parasite burden, as determined by qPCR. Parasite burden was highest at the heart base (position A) for strain CL and central heart segments (position C) for strain Sylvio X10/4, indicating parasite strain-specific differences in parasite tropism. (B) Statistically significant perturbations in the overall metabolite profile between uninfected and strain CL-infected mice (left), and between uninfected and strain Sylvio X10/4-infected mice (right). The highest significant metabolite perturbation was at central heart segments (position C) for strain CL (***, p < 0.001 by PERMANOVA) and at the heart apex (position D) for strain Sylvio X10/4 (**, p < 0.01 by PERMANOVA).

We observed a clear localized impact of *T*. *cruzi* infection on the overall metabolite profile (Figs 1, S2 and S3). As previously described [9], parasite burden was highest at the base of the heart (position A) for strain CL and central positions (position C) for strain Sylvio X10/4 (Fig 1A). PERMANOVA analysis indicated that the highest significant perturbation in the overall metabolite profile occurred at central positions for strain CL infection (PERMANOVA analysis of Bray-Curtis-Faith distance matrix R^2^ = 0.20813, p-value = 0.004 at position C) and at apical positions for strain Sylvio X10/4 infection (PERMANOVA R^2^ = 0.27923, p-value = 0.014 at position D) (Fig 1B). Strikingly, in both cases chemical disturbance was greatest at sites distinct from the site of highest parasite burden, which corroborates our observations in the context of chronic gastrointestinal *T*. *cruzi* infection in mice [18]. The localization of chemical disturbance also provides a molecular mechanism explaining the apical aneurysms observed in CD patients and the fact that these happen even though cardiac tissue parasite burden is low [22].

**Fig 2:**
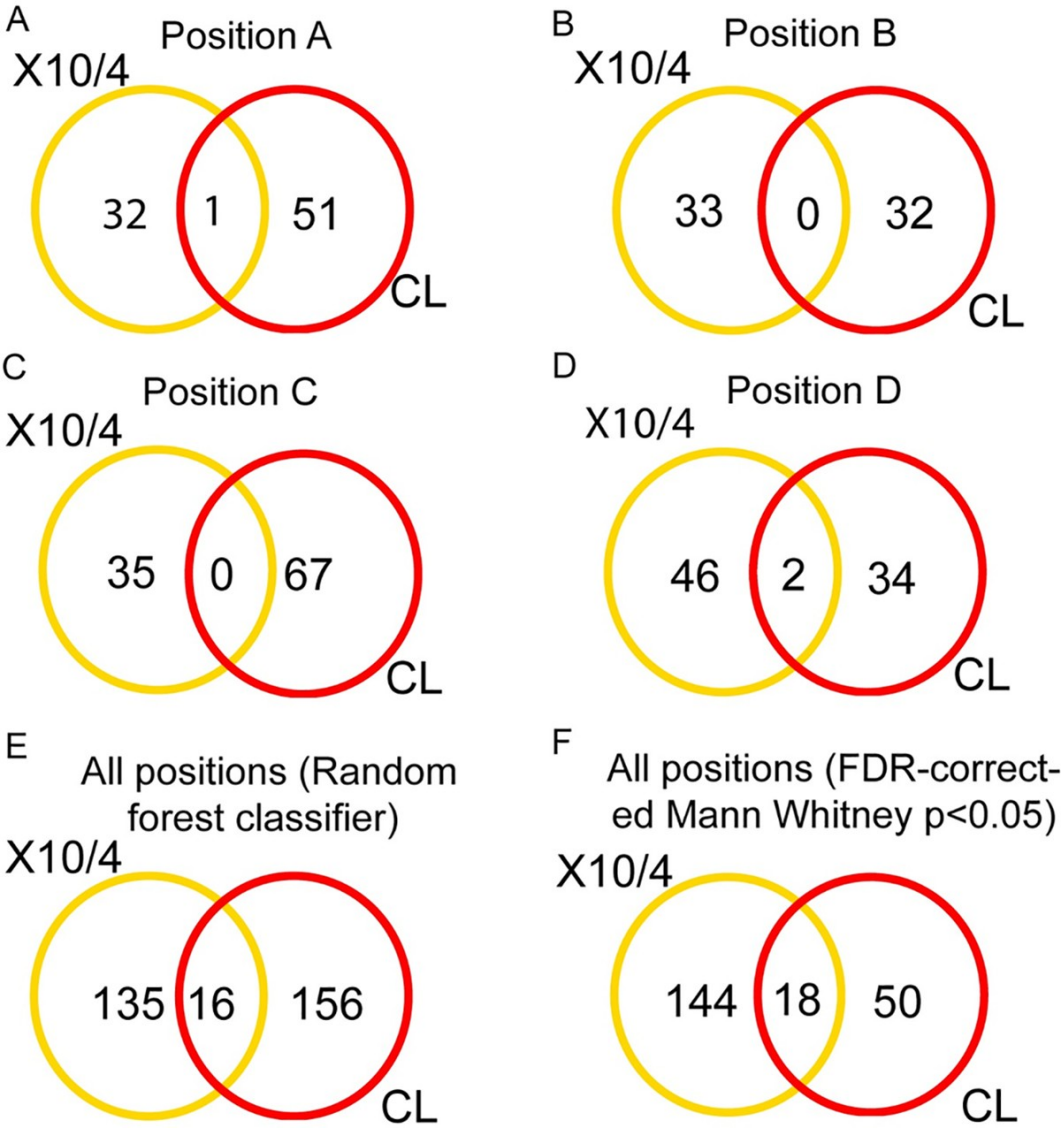
Limited overlap of specific differential metabolites between strains. Yellow and red circles represent differential metabolites between strain Sylvio X10/4-infected and matched uninfected controls, and between strain CL-infected and matched uninfected controls, respectively. Intersect are metabolites impacted by infection in both strains. (A-D) Differential metabolites for each strain, at given heart positions, as determined by random forest classifier, with variable importance score cutoff as described in Methods. (E) Metabolites impacted by infection with each strain, irrespective of position, as determined by random forest classifier, with variable importance score cutoff as described in Methods. (F) Metabolites impacted by infection with each strain, irrespective of heart position, using FDR-corrected Mann Whitney p<0.05 cutoff.

To identify the specific cardiac metabolites spatially perturbed by infection, initially we built a random forest classifier for each position, each strain and each extraction method, comparing to uninfected matched control samples (S1–S4 Tables). We first assessed the overlap between the top-ranked most differential metabolites by random forest for the two different strains, at each position, as described in Methods. Limited overlap of these significant metabolites was observed between strains (Fig 2A–2D). To address the possibility that a given metabolite was modified by each strain at different positions, we also performed this analysis for all positions combined (Fig 2E and 2F, S5 and S6 Tables). Indeed, a higher overlap was observed between strains under these conditions, but still only representing a fraction of all differential metabolites. However, annotation of these differential metabolites using molecular networking through the GNPS platform [30] revealed that while differing in terms of *m/z*, many were part of the same chemical families, including acylcarnitines and glycerophosphocholines (S1–S6 Tables, S4 and S5 Figs).

Random forest classifier identified several acylcarnitines and glycerophosphocholines as impacted by infection (S1–S5 Tables). Both chemical families play a major role in several biochemical pathways. Carnitine serves as a shuttling mechanism for fatty acids, in the form of acylcarnitines, from the cytosol into the matrix of the mitochondria for beta-oxidation [38]. Glycerophosphocholines are major components of lipid metabolism, cell membrane structure, and choline production, the latter of which is essential for select amino acid and neurotransmitter synthesis [39,40].

**Fig 3:**
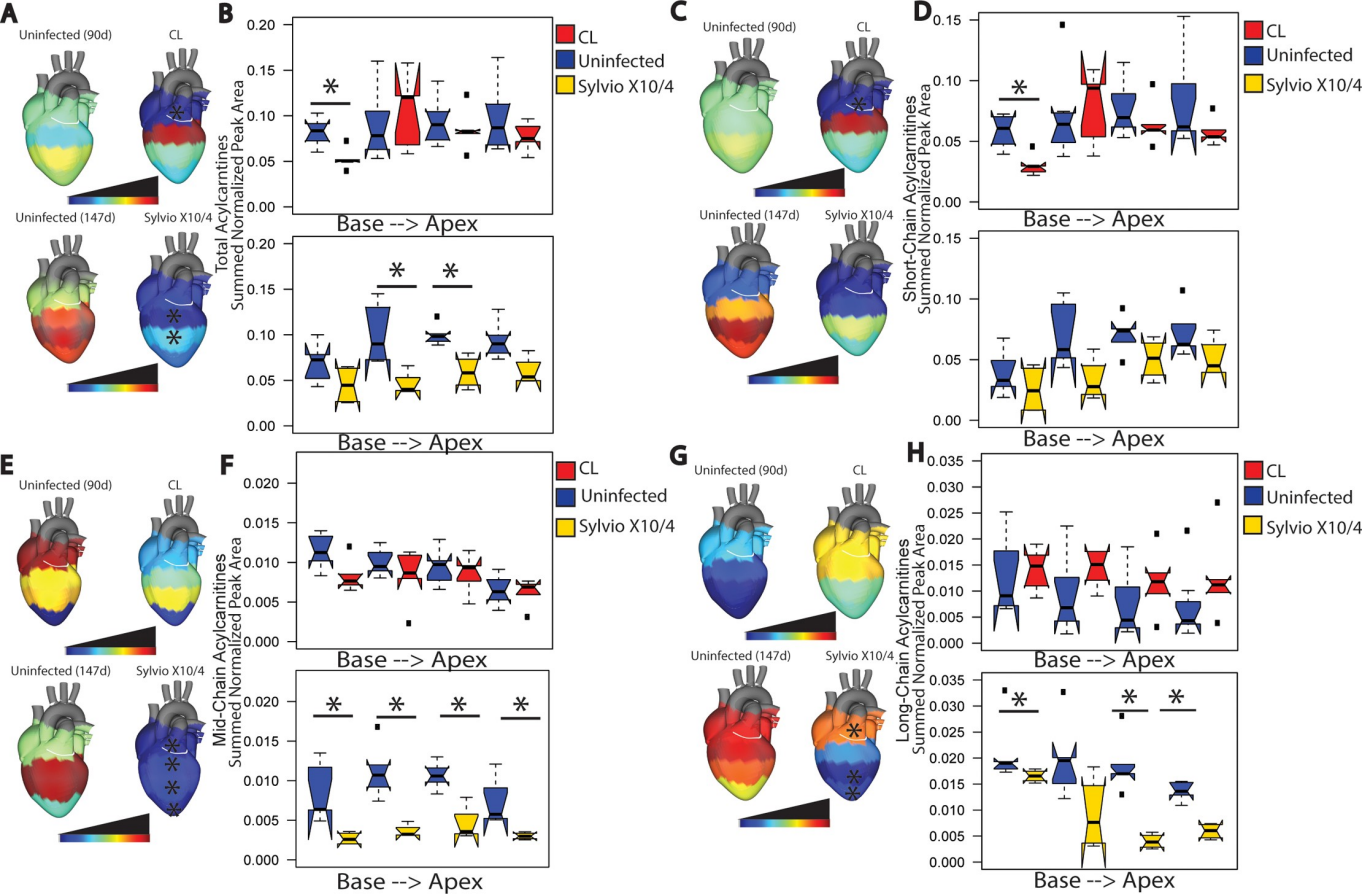
Spatial impact of chronic *T*. *cruzi* infection on cardiac acylcarnitines. Normal levels and distribution of acylcarnitines are represented by uninfected samples. (A, B) Differential total acylcarnitine distribution between uninfected and infected heart sections for both CL and Sylvio X10/4 strains. CL-infected mice showed statistically significant decreases in total acylcarnitine levels at heart base when compared to uninfected mice (*, p<0.05 by Mann-Whitney test). (C, D) CL-infected mice showed statistically significant decreases (*, p<0.05, by Mann-Whitney test) in short-chain acylcarnitine (≤ C4) at heart base. (E, F) Sylvio X10/4-infected mice showed statistically significant decreases in mid-chain acylcarnitines at all positions compared to uninfected mice. (*, p<0.05 by Mann-Whitney test). (G, H) Sylvio X10/4-infected mice showed statistically significant decreases in long-chain acylcarnitines (≥C12) at most heart positions compared to uninfected mice (*, p<0.05 by Mann-Whitney test).

Total acylcarnitines in the central positions of the heart were decreased by strain Sylvio X10/4 infection compared to the uninfected group (Fig 3A and 3B, Mann-Whitney p<0.05). A similar pattern was observed for total acylcarnitines following strain CL infection when compared to matched uninfected samples at the heart base (Fig 3A and 3B, Mann-Whitney p<0.05). Normal levels and distributions of acylcarnitines in the heart are represented by uninfected samples (Fig 3). Previous studies demonstrated that acylcarnitines of different lengths were associated with infection outcome in acute (12 days post-infection) *T*. *cruzi* mouse models [9]. Therefore, we sought to understand how different length acylcarnitines were affected by chronic (90 and 147 days post-infection) infection. Acylcarnitines are classified based on the number of carbons in their fatty acid chain as short- (≤C4), mid- (C5 -C11*)*, and long-chain (≥C12) acylcarnitines. In the case of CL strain infection, when compared to uninfected samples, short chain acylcarnitines were significantly decreased at the heart base (p<0.05 Mann-Whitney, Fig 3C and 3D). Strain Sylvio X10/4 infection significantly decreased mid-chain acylcarnitine at all positions (Mann-Whitney p<0.05, Fig 3E and 3F) and significantly decreased long-chain acylcarnitines at heart base, center and apex (Mann-Whitney p<0.05, Fig 3E–3H).

**Fig 4:**
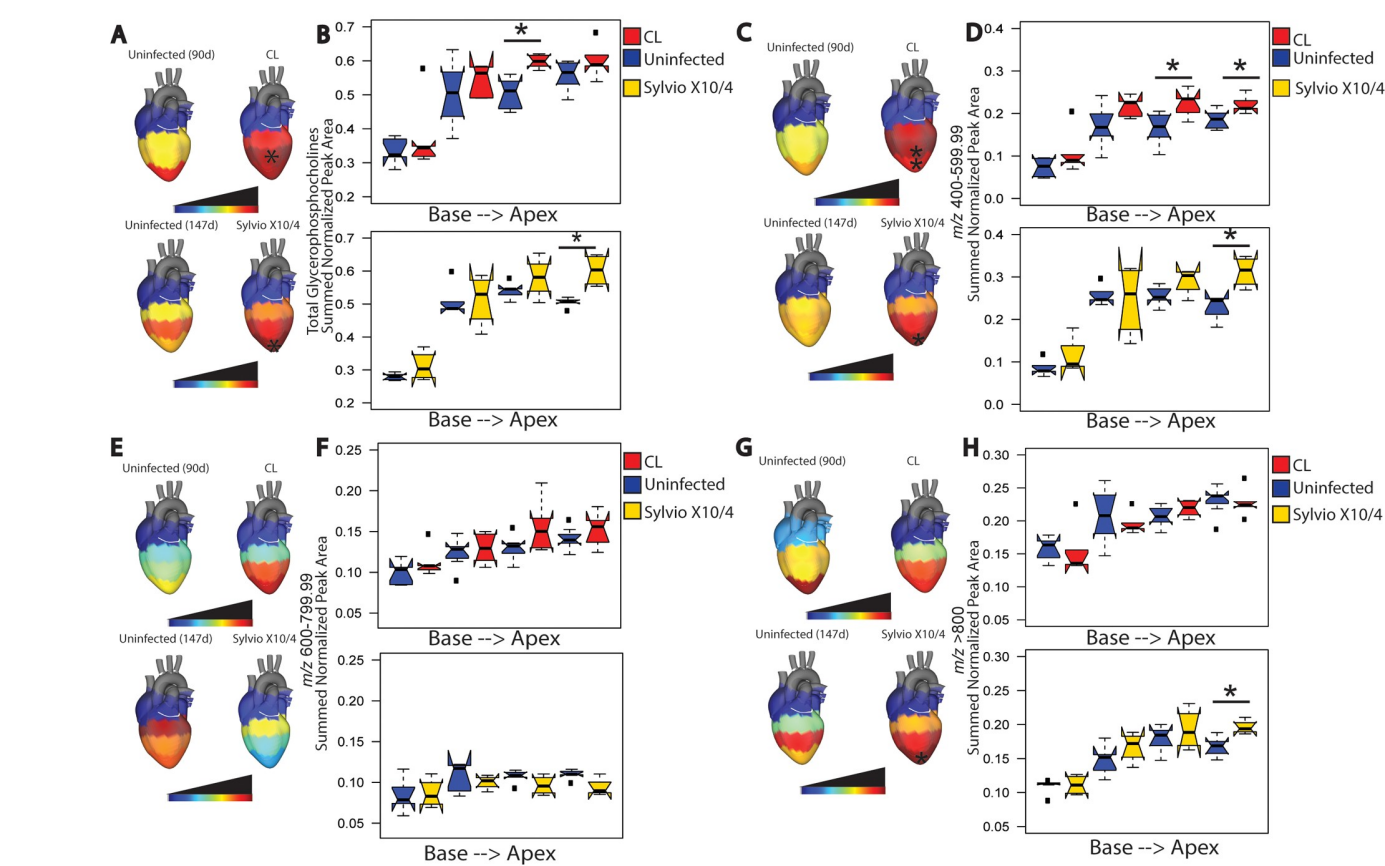
Spatial impact of chronic *T*. *cruzi* infection on cardiac glycerophosphocholines. (A, B) Differential total glycerophosphocholine distribution between uninfected and infected heart sections for both CL and Sylvio X10/4 strains. CL-infected mice showed statistically significant increases in total glycerophosphocholine levels at central heart positions when compared to uninfected mice (*, p<0.05 by Mann-Whitney test). (C,D) Both infected strains showed statistically significant increases (*, p<0.05, by Mann-Whitney test) for short glycerophosphocholines (*m/z* 400–599.99) at central and apical positions for strain CL and apical positions for strain Sylvio X10/4. (E, F) Mid-sized glycerophosphocholines (*m/z* 600–799.99) were not significantly affected by infection for both strains. (G, H) Sylvio X10/4-infected mice showed a statistically significant increase (*, p<0.05 by Mann-Whitney test) in long glycerophosphocholines (*m/z*> 800) at apical positions.

Select glycerophosphocholines were increased at specific sites upon infection. CL strain infection significantly increased total glycerophosphocholines, at central heart positions compared to uninfected samples (Mann-Whitney p<0.05), as did strain Sylvio X10/4 at the heart apex (Mann-Whitney p<0.05, Fig 4A and 4B). Further analysis based on glycerophosphocholine *m/z* was performed, because previous studies showed differences in glycerophosphocholine *m/z* range between fatal and non-fatal acute mouse infection [9]. Glycerophosphocholines were categorized into three mass ranges: short (400–599.99 *m/z*), mid (600–799.99 *m/z*), and long (>800 *m/z*). Significantly increased glycerophosphocholines were observed for CL strain infection in short (*m/z* 401–599.99) glycerophosphocholines at heart center and apex (Mann-Whitney p<0.05, Fig 4C and 4D). Sylvio X10/4 strain infection significantly increased short (*m/z* 401–599.99) and long (*m/z*>800) glycerophosphocholines at the heart apex when compared to uninfected samples (Mann-Whitney p<0.05, Fig 4C, 4D, 4G and 4H).

## Discussion

There are 7 *T*. *cruzi* discrete typing units infectious to humans (DTUs TcI—TcVI and Tcbat). These DTUs, while still currently considered the same species, nevertheless present significant genetic differences [41,42]. Select *T*. *cruzi* strains are also more virulent than others or require a greater dose to establish infection. The inocula used in this study for both strains are standard and based on their relative degree of acute-stage lethality [9,43]. Specifically, strain Sylvio X10/4 is a low virulence strain, necessitating a higher infectious inoculum to observe cardiac pathology at chronic timepoints [43]. In contrast, susceptible mice infected with 1000 CL trypomastigotes survive the acute stage to develop chronic cardiac symptoms, while mice infected with higher inocula only survive a few weeks [9]. Timing and magnitude of induced disease may also differ between *T*. *cruzi* strains. Ninety days timepoint for strain CL infection enables comparison with Hossain *et al*, which analyzed impact of infection on the gastrointestinal metabolome at 89 days post-infection [18]. In addition, the different doses and timepoints enabled us to find commonalities across infection systems, mimicking the clinical situation where patients do not know how long ago they were infected, or with which strain. Cross-strain, -dose and -timepoint comparisons are thus important to guide drug development.

However, pathogenic processes are overall similar in cardiac CD across *T*. *cruzi* strains, with accumulation of fibrosis and inflammation [42,44]. These similarities are reflected in the common metabolomic changes observed for strain Sylvio X10/4 (TcI) and strain CL (TcVI)-infected heart tissue in this study, including chronic infection-induced increases in glycerophosphocholines and decreases in acylcarnitines. These results concur with independent findings with regards to select acylcarnitines in the serum of CD1 mice chronically infected with *T*. *cruzi* strain Brazil (TcI) and to short-chain acylcarnitines and glycerophosphocholines in the heart of BALB/c mice acutely infected with *T*. *cruzi* strain Y (TcII) [15]. They also concur with the negative relationship between mid-chain acylcarnitines vs fibrosis and disease progression-associated cytokine PDGF, and the positive relationship between glycerophosphocholines in mass ranges 400–499, 500–599 and over all mass ranges vis-à-vis of inflammation, fibrosis and progression-associated cytokines in the heart of BALB/c mice chronically infected with *T*. *cruzi* strain H1 (TcI) [45].

Differences in pathogenesis between strains may be due to differential strain tropism. Indeed, TcI strains tend to produce cardiomyopathy, while TcVI strains commonly produce megacolon and megaesophagus, although cardiomyopathy can still occur [46]. Our results indicate a disconnect between sites of highest parasite burden and sites of metabolic perturbation. Although parasite levels were highest in central heart segments following strain Sylvio X10/4 infection, we observed statistically significant perturbations in metabolism at the apex of the heart (Fig 1). Apical aneurysms are one of the major symptoms in chronic CD patients [47]. In addition, lateral heart wall damage is also common among chronic CD patients, in central regions of the heart [48], and we observed significant perturbations in cardiac metabolism at lower central heart positions in strain CL infection (Fig 1B) [48]. These results are consistent with clinical findings of low cardiac tissue parasite burden in CD in humans. Although parasite persistence is required for Chagas disease progression, nevertheless, cardiac tissue parasite load was not correlated with the magnitude of tissue damage, in patient-derived samples [22]. Based on these results, we propose a concept of spatial disease tolerance, whereby some tissue regions are more affected by infection, while others are less functionally affected. This is likely due to a combination of host and pathogen factors, given the differences we observe here between strain CL and strain Sylvio X10/4 infection in the same C3H mouse genetic background. Importantly, the localization of maximal metabolic perturbation in acute strain Sylvio X10/4 infection was also the heart apex, indicating that the spatial course of disease may be set early in CD [9]. Likewise, host factors likely contribute, such as the higher production of antiparasitic but tissue-damaging IFNγ at the heart apex or specific cardiac regions being more prone to microvasculature disruptions [9].

Our results also highlight the importance of considering metabolic changes at the level of chemical families, beyond just individual metabolites. While there was little overlap of highly significant metabolite *m/z* at each position between strains, most differential annotatable metabolites were from these two chemical families. McCall *et al*. described these two chemical families as discriminatory compounds between fatal and non-fatal acute *T*. *cruzi* infected heart tissue [9]. Considering acute stage infection progresses into chronic stage infection, it is not surprising that changes in the relative abundance of these molecules are also observed in chronic CD.

Glycerophosphocholines have been linked to coronary heart disease due to production of lysophosphatidylcholines and choline [39,49]. Increased acylcarnitine levels have been linked to cardiovascular disease as well as cardiac symptoms in non-CD cardiac disease [50,51]. However, our results show the opposite pattern for acylcarnitines compared to non-infectious heart disease, highlighting the need to specifically study CD rather than extrapolate from other cardiac conditions. Indeed, this divergence was also observed at the level of gene expression profiles: upregulation of lipid metabolism gene expression was observed in heart samples of human cardiac CD patients compared to controls, while downregulation was seen in non-infectious dilated cardiomyopathy patient samples [52]. Higher lipid metabolism would increase acylcarnitine catabolism and thus decrease overall acylcarnitine abundance. Decreased carnitine palmitoyltransferase and acetyltransferase levels, as observed by proteomic analysis of infected mouse heart tissue [53], may alternatively also contribute to the decreased acylcarnitine levels we observed. Interestingly, a few infection-perturbed metabolites were annotated as odd chain saturated fatty acids. While rare, odd chain saturated fatty acids have also been linked to protection from coronary heart disease, atherosclerosis and type II diabetes [54–56].

These results set a foundation for host-directed therapeutic development. CD may be particularly amenable to such treatment strategies, due to the contribution of host-mediated tissue damage to CD pathogenesis [1,8]. Indeed, we have previously shown that carnitine supplementation can be used to treat acute CD [18]. These findings also demonstrate a causal role in disease pathogenesis for the metabolic alterations observed in our studies. Our observation of decreases in cardiac acylcarnitines in chronic CD indicate that carnitine-based treatment regimens may also be useful to treat chronic CD. Importantly, the fact that acylcarnitines are affected in both chronic CL and Sylvio X10/4 infection suggests cross-strain applicability. Other studies have emphasized the impact of metabolism modulators on CD progression. High fat diet reduces parasite levels and increases survival in acute CD mouse models [57]. Treatment of acutely *T*. *cruzi* infected mice with metformin (a metabolic modulator used to treat diabetic patients) also led to an increase in overall survival rate and decreased blood parasitemia [58].

Due to the low parasite burden in chronic Chagas disease and instrumental limits of detection, we anticipate most if not all detected metabolites to be host-derived, supported by their detection in uninfected tissues. As such, this study is focused on the impact of *T*. *cruzi* infection on host metabolism. A further limitation is that many of the differential metabolites were not annotatable, as is usual in metabolomic studies [59]. Nevertheless, we were able to annotate metabolites affected by chronic infection that make up important host biochemical pathways. We only analyzed samples in positive mode due to the greater availability of positive mode reference libraries, leading to better annotation rates (35.4% in positive mode vs 10.2% in negative mode [18]). A further limitation is that free fatty acids do not ionize well under our instrument conditions, so that we cannot link the changes in PCs to other lipids [60]. Lastly, effect size calculations on our total acylcarnitine and glycerophosphocholine data indicate that we were only adequately powered to detect large differences (Hedges’ g >0.6–0.8).

Overall, our study highlights the importance of identifying overall differences but also positional metabolic differences associated with infection, and the need to study multiple *T*. *cruzi* strains. Results also show the strength of systematic chemical cartography in understanding disease tropism and how it differs from pathogen tropism. These results will serve as stepping stones for further CD drug development, something that is urgently needed.

## Supporting information

S1 Table Annotated and unannotated metabolites perturbed by infection at position A, identified through random forest classifier. * indicate confidence level 3 annotations. All other annotations at level 2 confidence. NA, not annotated/not applicable.
(XLSX)

S2 Table Annotated and unannotated metabolites perturbed by infection at position B, identified through random forest classifier. * indicate confidence level 3 annotations. All other annotations at level 2 confidence. NA, not annotated/not applicable.
(XLSX)

S3 Table Annotated and unannotated metabolites perturbed by infection at position C, identified through random forest classifier. * indicate confidence level 3 annotations. All other annotations at level 2 confidence. NA, not annotated/not applicable.
(XLSX)

S4 Table Annotated and unannotated metabolites perturbed by infection at position D, identified through random forest classifier. * indicate confidence level 3 annotations. All other annotations at level 2 confidence. NA, not annotated/not applicable.
(XLSX)

S5 Table Annotated and unannotated metabolites perturbed by infection at positions A-D, identified through random forest classifier. * indicate confidence level 3 annotations. All other annotations at level 2 confidence. NA, not annotated/not applicable.
(XLSX)

S6 Table Annotated and unannotated metabolites identified as perturbed by infection at all positions (FDR-corrected Mann Whitney p<0.05). * indicate confidence level 3 annotations. All other annotations at level 2 confidence. NA, not annotated/not applicable.
(XLSX)

S1 Fig Base peak chromatogram of checkmix solution used to monitor instrumental drift. (A) Base peak chromatogram of organic extract checkmix solution at beginning (red) and end (blue) of LC-MS analysis. (B) Base peak chromatogram of aqueous extract checkmix solution at beginning (blue) and end (red) of LC-MS analysis.
(TIF)

S2 Fig **Principal coordinate analysis plot of *T*. *cruzi* strain CL infected (red) and uninfected (blue) heart tissue samples.** Statistically different clustering found in position C (PERMANOVA p-value<0.05).
(TIF)

S3 Fig **Principal coordinate analysis plot of *T*. *cruzi* strain Sylvio X10/4 infected (gold) and uninfected (blue) heart tissue samples.** Statistically different clustering found in position D (PERMANOVA p-value<0.05).
(TIF)

S4 Fig Molecular subnetworks and mirror plot of aqueous and organic extract acylcarnitines and glycerophosphocholines. Each pie chart is one metabolite colored by MS2 spectral count in CL-infected and Sylvio X10/4-infected samples where red is CL and gold is Sylvio X10/4. (A) Subnetwork of aqueous extract acylcarnitines with representative acylcarnitine mirror plot (acetylcarnitine, *m/z* 204.124). (B) Subnetwork of aqueous extract phosphocholines with representative glycerophosphocholine mirror plot (Spectral match to 1-Hexadecanoyl-2-(9Z-octadecenoyl)-sn-glycero-3-phosphocholine reference library spectrum, *m/z* 772.549). (C) Subnetwork of organic extract acylcarnitines with representative acylcarnitine mirror plot (palmitoylcarnitine, *m/z* 424.343). (D) Subnetwork of organic extract phosphocholines with representative glycerophosphocholine mirror plot (Spectral Match to 1-Stearoyl-2-linoleoyl-sn-glycero-3-phosphocholine reference library spectrum, *m/z* 794.57).
(TIF)

S5 Fig Representative GNPS mirror plots of annotated metabolites. (A) Mirror plot of *m/z* 384.116, RT 136s (top, black) to reference library spectrum (Succinyladenosine, bottom, green). (B) Mirror plot of *m/z* 137.047, RT 30s (top, black) to reference library spectrum (Hypoxanthine, bottom, green). (C) Mirror plot of *m/z* 148.061, RT 27s (top, black) to reference library spectrum (L-Glutamine, bottom, green). (D) Mirror plot of *m/z* 153.043, RT 34s (top, black) to reference library spectrum (Xanthine, bottom, green). (E) Mirror plot of *m/z* 538.52, RT 362s (top, black) to reference library spectrum (N-(1,3-dihydroxyoctadec-4-en-2-yl)tetradecanamide, bottom, green). (F) Mirror plot of *m/z* 657.204, RT 180s (top, black) to reference library spectrum (hemin cation, bottom, green).
(TIF)
