# Supplementary figures and images for: Spatial metabolomics identifies localized chemical changes in heart tissue during chronic cardiac Chagas Disease
Source: PLoS Negl Trop Dis. 2021 Oct 4;15(10):e0009819. doi: 10.1371/journal.pntd.0009819 (PMC8516257; doi:10.1371/journal.pntd.0009819)

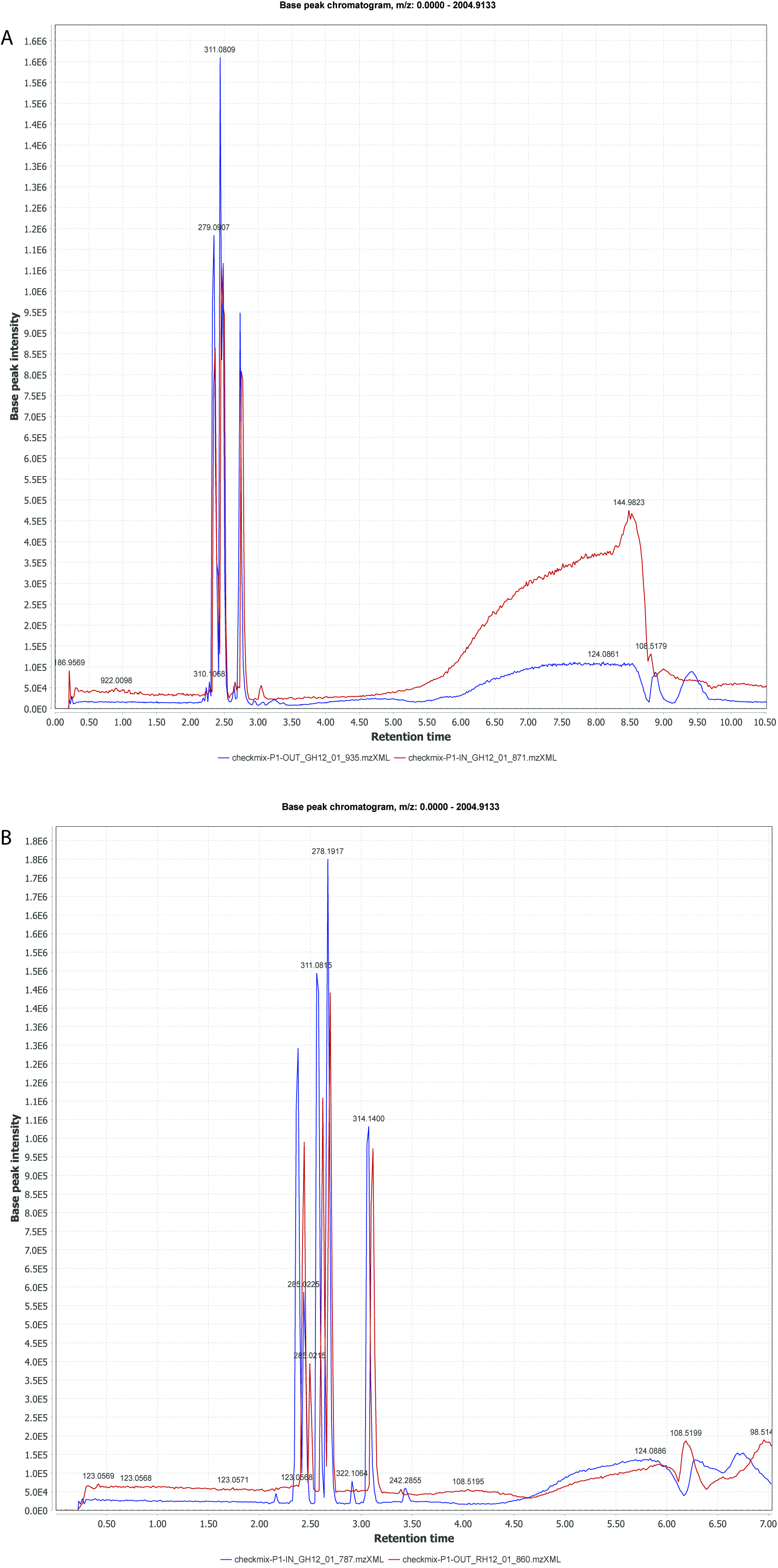

Supplement: S1 Fig — (A) Base peak chromatogram of organic extract checkmix solution at beginning (red) and end (blue) of LC-MS analysis. (B) Base peak chromatogram of aqueous extract checkmix solution at beginning (blue) and end (red) of LC-MS analysis. (TIF) [file pntd.0009819.s007.tif]

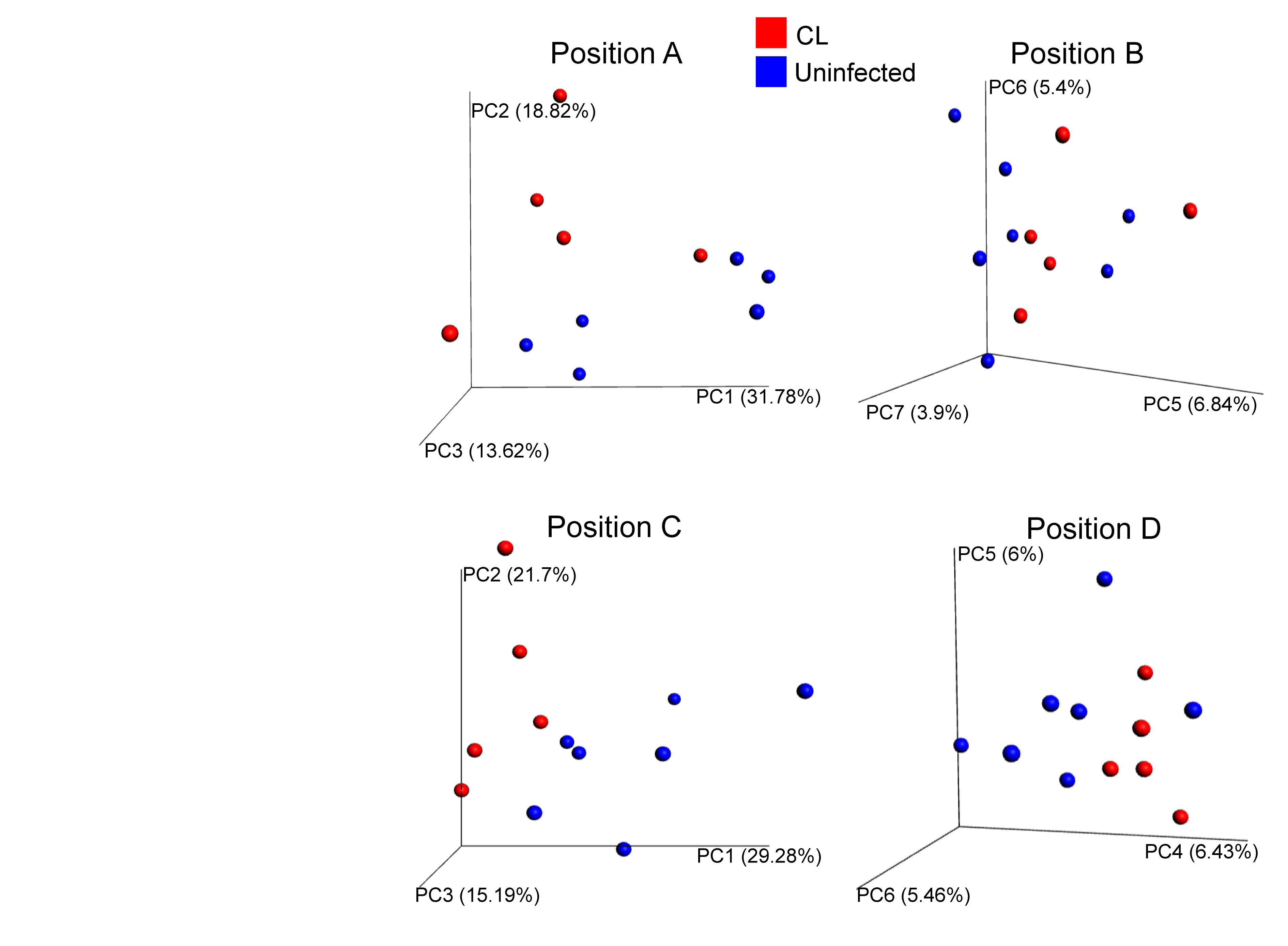

Supplement: S2 Fig — Principal coordinate analysis plot of T. cruzi strain CL infected (red) and uninfected (blue) heart tissue samples. Statistically different clustering found in position C (PERMANOVA p-value<0.05). (TIF) [file pntd.0009819.s008.tif]

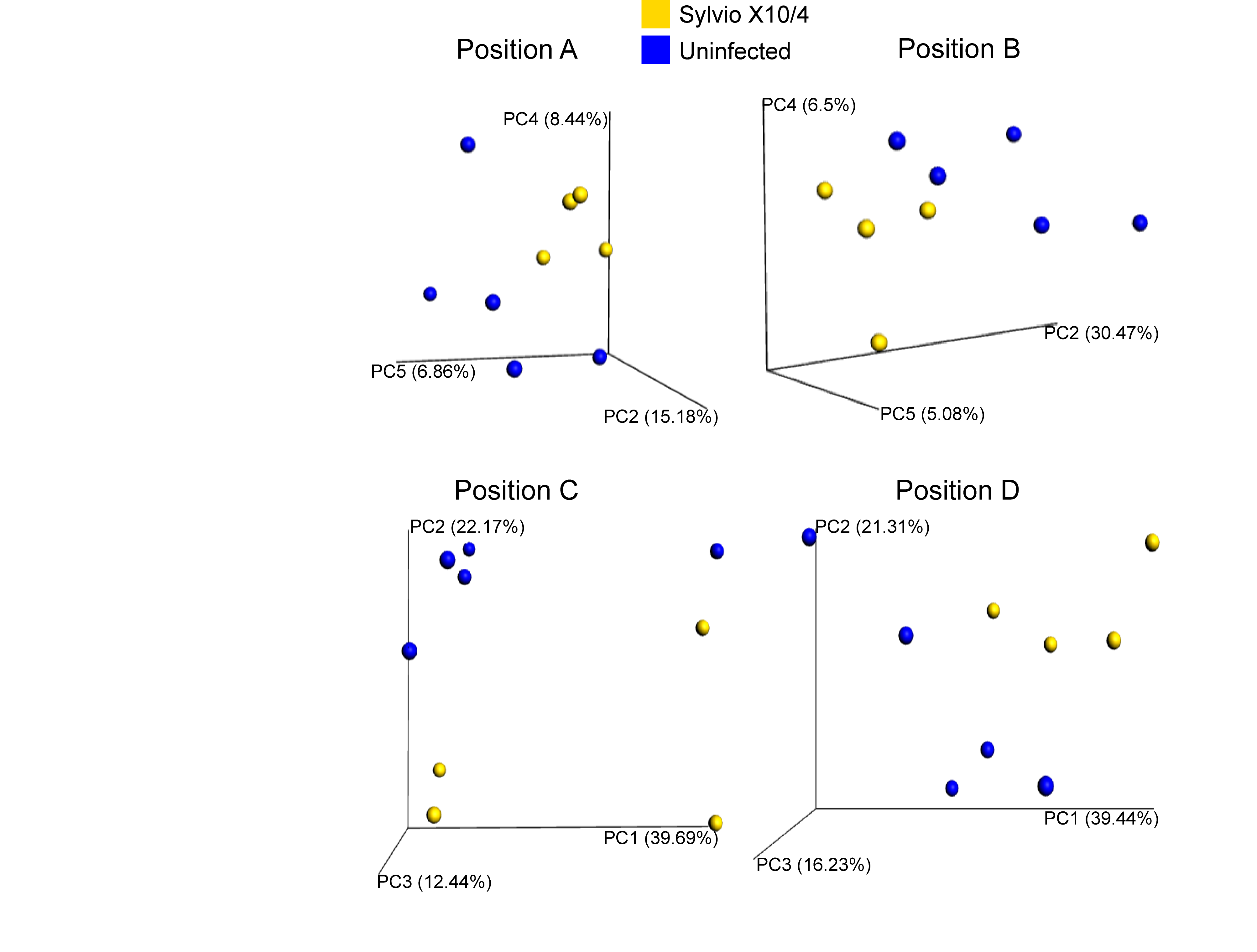

Supplement: S3 Fig — Principal coordinate analysis plot of T. cruzi strain Sylvio X10/4 infected (gold) and uninfected (blue) heart tissue samples. Statistically different clustering found in position D (PERMANOVA p-value<0.05). (TIF) [file pntd.0009819.s009.tif]

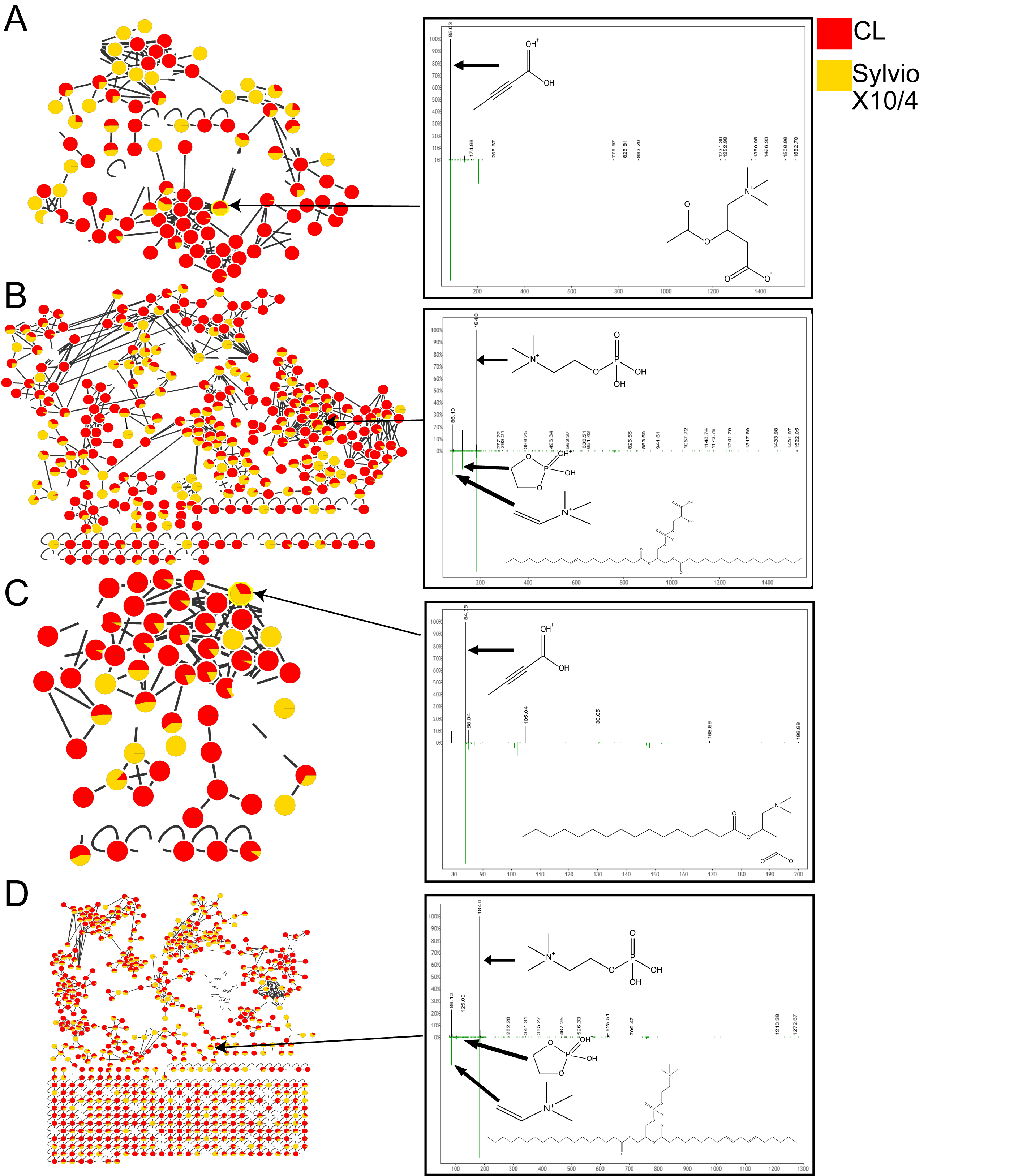

Supplement: S4 Fig — Each pie chart is one metabolite colored by MS2 spectral count in CL-infected and Sylvio X10/4-infected samples where red is CL and gold is Sylvio X10/4. (A) Subnetwork of aqueous extract acylcarnitines with representative acylcarnitine mirror plot (acetylcarnitine, m/z 204.124). (B) Subnetwork of aqueous extract phosphocholines with representative glycerophosphocholine mirror plot (Spectral match to 1-Hexadecanoyl-2-(9Z-octadecenoyl)-sn-glycero-3-phosphocholine reference library spectrum, m/z 772.549). (C) Subnetwork of organic extract acylcarnitines with representative acylcarnitine mirror plot (palmitoylcarnitine, m/z 424.343). (D) Subnetwork of organic extract phosphocholines with representative glycerophosphocholine mirror plot (Spectral Match to 1-Stearoyl-2-linoleoyl-sn-glycero-3-phosphocholine reference library spectrum, m/z 794.57). (TIF) [file pntd.0009819.s010.tif]

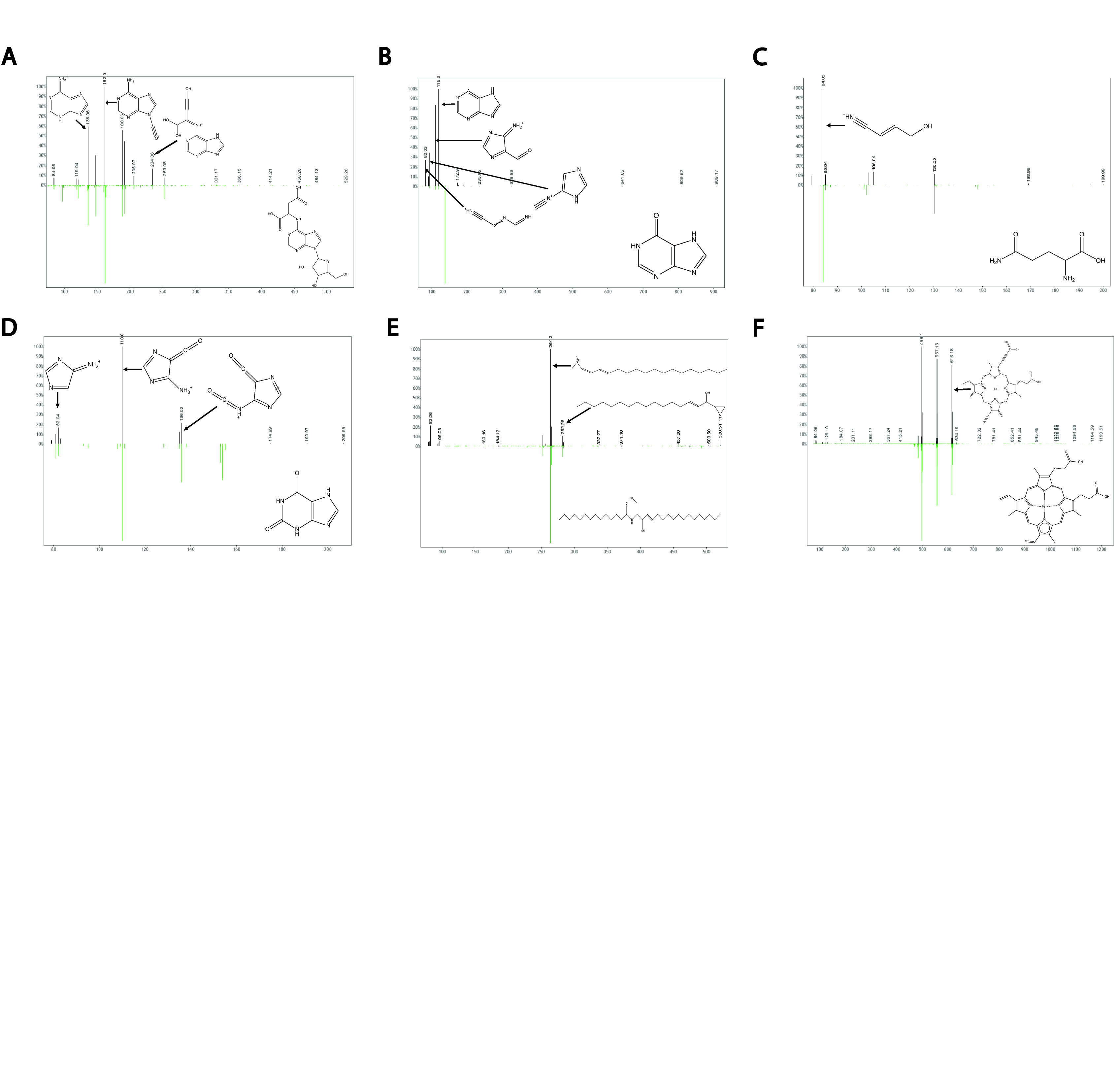

Supplement: S5 Fig — (A) Mirror plot of m/z 384.116, RT 136s (top, black) to reference library spectrum (Succinyladenosine, bottom, green). (B) Mirror plot of m/z 137.047, RT 30s (top, black) to reference library spectrum (Hypoxanthine, bottom, green). (C) Mirror plot of m/z 148.061, RT 27s (top, black) to reference library spectrum (L-Glutamine, bottom, green). (D) Mirror plot of m/z 153.043, RT 34s (top, black) to reference library spectrum (Xanthine, bottom, green). (E) Mirror plot of m/z 538.52, RT 362s (top, black) to reference library spectrum (N-(1,3-dihydroxyoctadec-4-en-2-yl)tetradecanamide, bottom, green). (F) Mirror plot of m/z 657.204, RT 180s (top, black) to reference library spectrum (hemin cation, bottom, green). (TIF) [file pntd.0009819.s011.tif]
